# Shelter-based convalescence for homeless adults in Amsterdam: a descriptive study

**DOI:** 10.1186/1472-6963-9-208

**Published:** 2009-11-18

**Authors:** Igor van Laere, Matty de Wit, Niek Klazinga

**Affiliations:** 1 Dr Valckenier Outreach Practice for Homeless People, GGD Municipal Public Health Service, PO Box 2200, 1000 CE Amsterdam, The Netherlands; 2 Department of Epidemiology, Documentation and Health Promotion, GGD Municipal Public Health Service, PO Box 2200, 1000 CE Amsterdam, The Netherlands; 3 Department of Social Medicine, Academic Medical Centre, PO Box 22660, 1100 DD Amsterdam, The Netherlands

## Abstract

**Background:**

Adequate support for homeless populations includes shelter and care to recuperate from illness and/or injury. This is a descriptive analysis of diagnoses and use of shelter-based convalescence in a cohort of homeless adults in Amsterdam.

**Methods:**

Demographics of ill homeless adults, diagnoses, referral pattern, length of stay, discharge locations, and mortality, were collected by treating physicians during outreach care provision in a shelter-based convalescence care facility in Amsterdam, from January 2001 through October 2007.

**Results:**

629 individuals accounted for 889 admissions to the convalescence care facility. 83% were male and 53% were born in the Netherlands. The mean age was 45 years (SD 10 years). The primary physical problems were skin disorders (37%), respiratory disorders (33%), digestive disorders (24%) and musculoskeletal disorders (21%). Common chronic conditions included addictions 78%, mental health disorders 20%, HIV/AIDS 11% and liver cirrhosis 5%. Referral sources were self-referred (18%), general hospitals (21%) and drug clinics (27%). The median length of stay was 20 days. After (self)discharge, 63% went back to the previous circumstances, 10% obtained housing, and 23% went to a medical or nursing setting. By March 2008, one in seven users (n = 83; 13%) were known to have died, the Standard Mortality Ratio was 7.5 (95% CI: 4.1-13.5). Over the years, fewer men were admitted, with significantly more self neglect, personality disorders and cocaine use. Lengths of stay increased significantly during the study period.

**Conclusion:**

Over the last years, the shelter-based convalescence care facility users were mainly homeless single males, around 45 years of age, with chronic problems due to substance use, mental health disorders and a frail physical condition, many of whom died a premature death. The facility has been flexible and responsive to the needs of the users and services available.

## Background

Over the last decades, shelter-based convalescence care programs, (also termed respite, infirmary, recuperative and intermediate care), increasingly emerged in the western world [1-13]. Programs differ from one another, though many provide room, board, on site 24-hours care, and a range of social and medical services. On average, these programs are small, with a median of 13 beds, and reimbursement depends on patchwork funding [6].

The limited body of research in Australia, Canada and the US suggests that these programs are cost-effective, reduce hospital readmissions, and have important social medical support and service-networking benefits for the clients [1-6]. However, it is argued that much remains to be learned about these programs, including their funding sources, their relationships and arrangements with hospitals and other referral sources, and where patients go when they are discharged from these programs [6].

To contribute to the knowledge, we describe a shelter-based convalescence program for ill homeless adults in Amsterdam, the Netherlands. A seven-year period of shelter-based convalescence use was reviewed to determine the demographics, medical diagnoses, referral patterns, length of stay, discharge locations, mortality rate, and use patterns. Information about the experiences in this specific shelter will help program and policy makers to design or adjust shelters services that adequately fit the needs of homeless populations, and are efficiently linked to the healthcare system.

### Shelter-based convalescence program in Amsterdam

In Amsterdam, shelter-based convalescence care facilities were introduced in the early 1990s. In these days a relatively small proportion of the Amsterdam general hospital beds were occupied by HIV infected drugs users [14]. As a result of lifestyle concerns and strict admissions criteria, aftercare for this group was not offered by the mainstream nursing homes. Initially in two shelters, a total of ten overnight beds were transformed to 24-hour convalescence care beds to fill the hospital-to-streets gap. Through the years, in response to a growing care need, in three shelters the number of convalescence beds has increased to a total of 134 beds today. The convalescence care beds were embedded in the system of medical care provided by health professionals from the Municipal Public Health Service (MPHS) in Amsterdam, that also provides outreach medical care in three day centres, and three overnight shelters and 18 residence shelters (in total 1,090 beds) [15,16]. At most sites, MPHS health workers have access to online electronic client medical health records. The client records aim to give an overview of the social medical care biography and relevant medical letters from healthcare providers in the care network, and the actual medication prescribed, network partners, and care plan.

The shelter-based convalescence care facilities are staffed by nurses, orderly, social workers, housekeepers, and volunteers and offer integrated and problem oriented services that include a bed, food, clothing, on site 24-hour nursing care, medication compliance by daily observed therapy, wound care, vaccinations, wheel chair access, physical therapy, assistance for identity cards, benefits, debt control and health insurance, family reunion, pastoral support, and transportation to relevant services. Shelter rules tell to behave and not to consume alcohol or drugs on premises. The costs for this service were covered by Amsterdam Welfare department payments per user per night, donations by the public, and a contribution for board, lodging, and health insurance preemies paid by the users.

Sources of referral are homeless people themselves, medical workers in the primary and secondary care sector, and by social workers, the police and penitentiary staff. Although most referrals occur during the day, for advice and/or admission the MPHS health workers can be contacted around the clock, all days of the year.

Criteria for admission are homelessness and ill health and/or injury, often in combination with chronic problems with addiction, mental and physical health. MPHS outreach physicians are responsible for the admission assessment, direct medical care, making the individual care plan and follow up. In case patients are too sick to stay they are transferred to general hospitals. Convalescence care users can be admitted up to three months. Based on interdisciplinary observations trajectories for suitable housing and care after discharge are initiated. The length of admission can be prolonged another three months, or longer for those with multiple conditions in need for chronic nursing care, palliative care for the terminally ill included [15].

## Methods

### Study population and data collection

For this study, data were collected at a shelter-based convalescence care facility, named the Gastenburgh, a Salvation Army run shelter located in the Amsterdam red-light district. It started as an overnight shelter in the 1980s, and gradually transformed into a facility with 25 convalescence care beds and 25 chronic nursing care beds today. At admission, the patient was assessed by the treating MPHS physician, and demographic data, medical conditions, medication and treatment plan were recorded. The experience of the treating physician [17], referral letters and the available medical letters in the MPHS electronic client records were used, and diagnoses were coded according the International Classification of Primary Care (ICPC) [18]. Data were collected for and during all admissions from January 2001 and October 2007. Patient consent was obtained at admission.

Referring partners in the care network included several outreach centres in locations throughout Amsterdam, and patients were self-referred and admitted for convalescence care. Referrals also occurred through social networks such as social workers at day centres and general residence shelters, police, and after release from prison. Medical referrals included those from general practitioners, hospitals, MPHS outreach safety net teams and MPHS drug clinics [16], as well as addiction health clinics and mental health services. The duration of the admissions was measured in days, from the date of admission till the date of discharge or death.

The whereabouts after discharge where divided in social and medical settings. Social settings could be: a house (rent apartment, sub renting, including doubling up with family or friends), general residence shelters, prison, the streets and unsheltered places, or unknown in case of self discharge or expulsion due to misconduct. Medical settings could be: a shelter-based chronic nursing care facility, nursing homes, hospitals, addiction- and mental health residence clinics. To determine the mortality rate, by patient name and date of birth, the Amsterdam population register and MPHS electronic patient records were used up till March 2008. The study design did not need a process of ethical approval according to the Dutch Act on Medical Research.

### Study assessments and analysis

Statistical analyses were performed using SPSS 14.0 and were mainly descriptive. Demographics, diagnoses, length of stay and whereabouts after discharge were compared between the years of admission. Differences were compared using chi-square and Fisher-exact tests for categorical variables and Wilcoxon median test for continuous variables. Trends over the years were tested with trend analyses. The mortality rate was calculated from time up of admission until death or until the end of follow-up (March 2008). The standard mortality ratio was calculated by comparing the mortality among the users with the mortality in a comparable group (5-year-age groups, gender, ethnic background) in the general Amsterdam population. Survival analysis was performed to determine factors independently associated with higher mortality rates.

## Results

Written consent for inclusion to access information was obtained, this was granted in 99% of those asked. With a total of 889 admissions by 629 unduplicated individuals, between January 2001 and October 2007, the majority of the convalescence care users were admitted once (75%) or twice (18%). A small group (n = 46) was admitted from 3-13 times for a total 192 admissions; this was 22% of all admissions. No seasonal influences were noticed, as 54% of the admissions were in October to March.

### Demographics and chronic medical conditions

In table 1, the demographics and chronic medical conditions are shown. Most were men, between 30-60 years old, and over half were born in the Netherlands and nearly one fifth in Surinam and the Netherlands Antilles (former Dutch colonies). The mean age was 45 years (SD 10.2 years). Among those entitled to a residence permit (n = 552), 32% did not have a health insurance. The mean age of the 86 illegal immigrants was 39.9 years, and eleven illegal immigrants were female. The younger group (<45 years) included relatively more females (p < 0.001) and illegal immigrants (p < 0.001). The older group (45 years and older) included relatively more users born in Surinam and the Netherlands Antilles (p < 0.001).

**Table 1:** Demographics and chronic medical conditions among shelter-based convalescence care users in Amsterdam between 2001-2007

*Demographics *(n = 629)	n	%
Male	520	83
Female	109	17
Age in years*		
18-29	36	6
30-39	163	26
40-49	220	35
50-59	153	24
60-78	57	9
Country of birth		
Netherlands	334	53
Surinam/Antilles	114	18
Morocco	36	6
Europe/North America	89	14
Africa/Asia/South America	56	9
Illegal immigrant	86	14
Health insurance (n = 552)**	364	68
*Chronic medical conditions*		
Addiction total (overlap)	493	78
heroine (and/or cocaine)	259	41
methadone prescription	254	39
cocaine (no heroine)	114	18
alcohol	176	28
Mental health disorder	131	21
Addiction and mental health disorder	83	13
HIV infection	72	11
Tuberculosis life time	32	5

* Mean age females 41.3 years (SD 9.2 years) (range 19-74 years); mean age males 45.8 years (SD 10.3 years) (range 18-78 years).

** In 2006, among the general Dutch male population (approx. 8 million citizens) 2.1% did not have a health insurance (Dutch Central Bureau of Statistics, 2007).

As expected, a high prevalence of addiction (78%) and mental health problems (21%) were encountered. Out of 259 heroin users, 95% were prescribed methadone, and the median dosage was fifty milligrams. Among 114 cocaine users, 26% also used alcohol and 25% had a coexisting mental illness. Heroine use was less common among the mentally ill (28% versus 45%, p = 0.001). Heroine users, most of whom were former injectors, were three times more often HIV infected than those not using heroin (19% versus 6%; p < 0.001).

### Diagnoses upon admission

In table 2, the medical diagnoses upon admission are shown. The most frequently noted diagnoses were psychological disorders (poor hygiene 47%, schizophrenia 5% and personality disorders 14%), skin disorders (immersion foot 17%, skin injuries and infections 13%, erysipelas 12%, and chronic ulcers 4%), respiratory disorders (pneumonia 22% and COPD 15%), digestive disorders (hepatitis B/C 11%, gastroenteritis 7% and cirrhosis of the liver 5%) and musculoskeletal disorders (injuries 19% and fractures 6%). Other diagnoses were exhaustion in 8%, diabetes in 7%, epilepsy in 5% and incontinence of urine in 4%. Thirteen individuals were diagnosed with a malignancy (2%). On average, 2.7 medical diagnoses were noted per admission.

**Table 2:** Medical diagnoses upon shelter-based convalescence admissions in Amsterdam between 2001-2007

		admissions		persons	
ICPC *	Chapter	n	%	n	%
P	psychological	541	61	380	60
S	skin	326	37	244	39
R	respiratory	296	33	212	34
D	digestive	215	24	180	29
L	musculoskeletal (locomotion)	188	21	165	26
K	circulatory	123	14	100	16
A	general and unspecified	114	13	101	16
T	endocrine, metabolic, nutritional	97	11	77	12
N	neurological	87	10	78	12
U	urological	46	5	37	6
B	blood, spleen, bone marrow	32	4	23	4
F	eye	15	2	14	2
X	female genital	13	2	12	2
Y	male genital	13	2	12	2
H	ear (hearing)	4	0.4	4	0.6
	total	889		629	

* International Classification of Primary Care (ICPC) [18].

### Referrals, length of stay and discharge locations

In table 3, referrals, length of stay and discharge locations are shown. The major referral sources were general hospitals and MPHS drug clinics. A large number of admissions had a length up to two weeks (41%). The median duration of admission was 20 days, the average length of stay was 47 days, ranging from self discharge within 24 hours to 811 days. After discharge the majority went back to the previous circumstances, such as the streets and overnight shelters. One tenth obtained housing in an apartment or general residence shelter. For 5% the condition had worsened and were transferred to a general hospital. Despite a high rate of addiction and mental health problems, only a few went to a residential clinic for these problems. Among those who had multiple problems and needed chronic and/or palliative care, 13% stayed for this in the Gastenburgh.

**Table 3:** Referrals, length of stay and discharge locations of shelter-based convalescence care users (n = 629) in Amsterdam between 2001-2007

*Referrals to convalescence care *(n = 889)	n	%
Self referral	163	18
Social referrals total	181	20
day centres/shelters	142	16
prison/police	39	4
Medical referrals total	545	61
general practitioners	68	8
general hospitals	188	21
MPHS *drug clinics/outreach teams	236	27
mental/addiction health clinics	53	6
*Length of stay *(days)		
0-14	361	41
15-30	169	19
30-90	234	26
90-120	43	5
> 120	82	9
*Discharge locations*		
Social setting	679	76
street/self discharge	326	37
overnight shelter	166	19
general shelter	50	6
apartment	39	4
went abroad	13	2
expelled after misconduct	28	3
prison	57	6
Medical setting	201	23
general hospital	40	5
shelter based chronic nursing care	115	13
general nursing home	13	2
mental/addiction health clinic	33	3
Died during admission	9	1

* Municipal Public Health Service, Amsterdam.

### Mortality

The Amsterdam population register and MPHS electronic patient records were analysed for all convalescence care users that had died between their admission and March 2008. Among 629 homeless users, 517 were known to the Amsterdam population register, illegal immigrants were not registered, and 83 were known to have died (13.2%). For one person the date of death was unknown. Among 82 deaths, 74 male, the mean age was 52.7 years (SD 10.7 years; range 32-77 years). The convalescence care users died seven and a half times more often than the general Amsterdam population with comparable sex and age. Overall, the standard mortality ratio was 7.5 (95% CI: 4.1-13.5), and the figures were 7.6 and 6.5 for males and females, respectively. Survival analysis, with correction for age and sex, showed an increased mortality risk for HIV, hazard ratio 3.5 (95% CI: 2.1-5.7); dual diagnosis 2.2 (95% CI: 1.3-3.9); cirrhosis of the liver, 2.1 (95% CI: 1.0-4.6); mental illness, 1.6 (95% CI: 1.0-2.6); and malignancy, 7.8 (95% CI: 3.5-17.2).

### Users pattern over seven years

In table 4, the users pattern and service data are shown. The group of convalescence care users became smaller and stayed significantly longer. The number of self-referrals decreased, referrals through social partners increased and less self-discharge was noted. Over the years, the percentage of males and those born in Surinam and the Netherlands Antilles increased significantly. A trend of more psychological problems was noticed, cocaine users were increasingly admitted and the number of HIV infected users tended to decrease.

**Table 4:** User profile and service data of shelter-based convalescence care users in Amsterdam between 2001-2007

	2001	2002	2003	2004	2005	2006	2007	total		P*
*user profile (n = 629)*	%	%	%	%	%	%	%	%	n	
male	75	77	86	81	87	89	83	81	724	**p = 0,020**
born in Surinam/Antilles	17	12	19	22	20	27	23	19	167	**p = 0.021**
psychological disorder	41	71	67	58	64	68	73	61	541	**p = 0.000**
skin disorder	35	43	37	28	41	50	35	37	326	p = 0.052
circulatory disorder	20	12	11	17	10	11	5	14	123	p = 0.060
cocaine addiction	11	22	21	20	16	34	38	20	180	**p = 0,000**
HIV	19	14	11	14	16	5	10	14	122	p = 0.061
*service data*										
self referrals	36	32	19	3	7	5	3	18	163	**p = 0,000**
social care referral	6	8	24	27	30	41	33	20	181	**p = 0,000**
self discharge/streets	41	47	48	36	42	18	23	41	363	**p = 0.001**
health insurance	81	70	65	57	55	80	78	67	517	p = 0.510
median length of stay (days)	15	17	16	34	20	101	(31)**	20	-	**p = 0.000**
absolute number of admissions	158	150	238	191	70	40	40	100	889	

* Linear-by-Linear Association.

** For 25 homeless adults the length of stay is cut off at the end of the study episode at October 1, 2007.

## Discussion

### Characteristics of users and admissions

This study analysed the profile and dynamics of shelter-based convalescence care users over a period of seven years in Amsterdam. The users were mainly male, around 45 years and Dutch born. Upon admission, the physical problems primarily consisted of disorders of the skin as well as pulmonary, digestive and musculoskeletal conditions. Chronic medical problems were mainly substance use (78%), mental illness (21%), HIV/AIDS (11%) and cirrhosis of the liver (5%). Referrals were interlinked with the services available, and general hospitals and MPHS drug clinics were the main sources. After an average stay of 47 days, only 10% improved their housing situation and 23% went to a medical setting. The overall mortality rate was 13%, and independent risk factors were male gender, HIV, mental illness, dual diagnosis, liver cirrhosis and malignancy. Over the years, fewer men were admitted, with significantly more self neglect, personality disorders and cocaine use. Lengths of stay increased and less self-discharge was noticed during the study period.

### Strengths and limitations

The strengths of this study are that the provision of outreach care and the collection of data were done by the same individuals. During the study period of seven years, data were collected systematically and the diagnoses, assigned in most cases by specialists in general hospitals and drug clinics, were scored by the same outreach physician. This study has several limitations. First, the sample was a selection of ill homeless people who were in contact with service providers and who knew the routing towards admission for convalescence care. Therefore, the data can not be generalised to the total ill homeless population, including those out of reach of services in Amsterdam. Second, underreporting of medical conditions is likely due to limitation of record distraction of often voluminous medical records, and due to unshared information among multiple medical service providers. Third, the mortality rate might be higher than reported here due to incomplete data in the population registrar and MPHS electronic patients records, e.g. death of unidentified corpses, loss to follow up, and illegal immigrants who are not included in the official death statistics.

### Comparison to other convalescence care facilities

In Australia, Canada, Germany, the Netherlands and the US, convalescence care users were predominantly male, and the mean age was also around 45 years. The race was mostly Caucasian in Australian, Canadian and Dutch studies while in the US most were African American [1-8]. The medical conditions stated in our study are comparable to other studies of convalescence care in homeless persons. In convalescence care studies the users presented, more or less, with what O'Connell et al. refer to as tri-morbidity: a mix of addiction, mental and physical health problems [19]. We found 59% drug users, 28% alcohol users and 21% were known with a mental illness. Among convalescence care users in Rotterdam (n = 99); the figures are similar; drugs 69%, alcohol 32% and mental illness 28% [8]. Among Cottage Project users in Melbourne (n = 45), the figures were; alcohol 70%, drugs 32%, and mental illness 14% [1]. In Canada and the US, the figures for substance use were 30% and 33% respectively, and for mental illness 84% and 46% respectively [2-4]. These figures, including physical problems, show a high prevalence of tri-morbidity among convalescence care users in the western world. Our referral patterns, length of stay and discharge locations are comparable to those in other studies, and discharge locations were, more or less, the previous circumstances, residence shelters, and facilities for chronic nursing or hospice care [1-8].

### Mortality

Thirteen percent of the users had died during the course of our study. In Boston, O'Connell et al.[19] designed a high risk profile among homeless people, based on risk factors for premature mortality among homeless persons, that sleep on the streets 6 months or longer with one of the following conditions: 1) tri-morbidity of substance use, severe persistent mental illness, and multiple chronic physical problems, 2) multiple physical problem(s) resulting in hospital admission, multiple emergency department visits (3 or more visits in the previous 3 months), or admission to the respite facility anytime during the previous year, 3) age over 60 years, 4) known HIV/AIDS, 5) known cirrhosis, end-stage liver disease or renal failure 6) previous history of frostbite, hypothermia, or immersion foot. These conditions are consistent with those among the homeless in our study. Many users were diagnosed with tri-morbidity, 21% stayed in a general hospital prior to convalescence care admission, all were admitted for convalescence care (1-13 times in 7 years), 9% was over 60 years, 11% was known to be HIV infected, 5% had liver cirrhosis and 17% presented immersion foot. Compatibly, we found an increased mortality risk for HIV, dual diagnosis, liver cirrhosis and mental illness.

The high mortality rate among the convalescence users in our study might be explained by the fact that the homeless population in Amsterdam most commonly consists of mentally ill people who would have been admitted in mental health institutions 20-30 years ago, and long-term opiate users and alcoholics who can not live independently, and who depend on fragmented services [15,16]. Furthermore, the Netherlands is an advanced welfare state with a large social housing sector, housing and welfare benefits, universal health insurance, and numerous arrangements for the lowest income groups. Those who fall through all safety nets available might be the most difficult to serve in the community.

### 15 years convalescence experience and practice implications

In our experience, referrals, admissions and destinations after discharge depend on many factors. What is the size and nature, and the development of the profile, of the homeless population and of the community services? Do homeless people themselves know when, how and where to find assistance? Are the partners in the mainstream social and medical care network aware of the existence of the convalescence service, the admission criteria and the routing to realise admission? Is transportation or personal guidance needed to make sure the ill homeless person will arrive at the shelter? Is payment or having a medical insurance card obligatory to access? Are the facility and staff equipped to address multiple and complex conditions [20,21]? Furthermore, the length of admission, hence the next place to stay, depends on the nature and severity of problems among the convalescence care users on one hand, and the availability of problem oriented services in the community on the other. Waiting lists for a place in a general shelter or guided living facilities extend the length of stay.

In Amsterdam, the development of the size and nature of marginalized populations, such as homeless people, drug users and mentally ill patients, has been monitored for many years [15,16]. We have been witnessing an aging and frail population in growing need for tri-morbidity and palliative care. Among the homeless population, a subgroup suffers extreme cocaine and/or alcohol dependence and conduct disorders that make them hard to serve other than during moments of crisis, and multiple hospital, convalescence and prison admissions. Over the recent years, however, to several individuals with this profile, compulsory psychiatric treatment measures have been applied to reduce harm and prevent avoidable deaths.

In anticipation to trends and care needs among homeless people in Amsterdam [22], and with substantial national and local financial support, housing, social and medical services have been able to expand their activities. More guided living options in the social housing sector are being offered, more integrated one stop social medical service units are and will be build, and the number of beds in shelters, addiction and mental health care facilities are being increased [23]. In addition, in 2003, the sheltered-based convalescence care facilities, as well as general shelters and regular nursing homes, were able to adapt and/or transform their services into a chronic guiding, nursing and/or convalescence facility, by additional public insurance funding through the Exceptional Medical Expenses Act. As a result, community services have been able to cater for more marginalised people. It is within this context, most likely, that we witnessed a decrease of the number of admissions, an increase in the length of stay and less self-discharge towards the end of our study. The convalescence facility has been flexible and responsive to the needs of the users and services available.

## Conclusion

In Amsterdam, community services are challenged to prevent homelessness most commonly among single living men with financial mismanagement, addictions and/or mental health problems [24-26]. Specifically, treatment services should target a new generation of cocaine users to prevent further marginalisation [27]. To reduce harm to the individual and society, care providers should target individuals at high risk of tri-morbidity and mortality. To apply upstream prevention strategies, intensive social medical care programs, similar to the nature of shelter-based convalescence programs, should be available continuously before and during homelessness.

## Competing interests

The authors declare they have no competing interests. No funding was provided for this research.

## Authors' contributions

IvL contributed to the study design and implementation, collected data and wrote the manuscript. MdW analysed the data and assisted in writing the manuscript. NK contributed to the manuscript design and assisted in writing the manuscript. All authors read and approved the final manuscript.

## Pre-publication history

The pre-publication history for this paper can be accessed here:

http://www.biomedcentral.com/1472-6963/9/208/prepub
